# *SORL1* gene, plasma biomarkers, and the risk of Alzheimer’s disease for the Han Chinese population in Taiwan

**DOI:** 10.1186/s13195-016-0222-x

**Published:** 2016-12-30

**Authors:** Cheng-Ta Chou, Yi-Chu Liao, Wei-Ju Lee, Shuu-Jiun Wang, Jong-Ling Fuh

**Affiliations:** 1 Department of Neurology, Neurological Institute, Taichung Veterans General Hospital, 1650 Taiwan Boulevard Section 4, Taichung, 40705 Taiwan; 2 Department of Neurology, Neurological Institute, Taipei Veterans General Hospital, No. 201, Section 2, Shipai Road, Beitou District, Taipei City, 11217 Taiwan; 3 Department of Neurology, Faculty of Medicine, National Yang-Ming University, No. 155, Section 2, Li-Nong Street, Taipei, 11217 Taiwan; 4 Institute of Clinical Medicine, National Yang-Ming University, No. 155, Section 2, Li-Nong Street, Taipei, 11217 Taiwan; 5 Brain Research Center, School of Medicine, National Yang-Ming University, No. 155, Section 2, Linong Street, Taipei, 11217 Taiwan

**Keywords:** *SORL1* gene, Amyloid-beta, Plasma biomarkers, Polymorphisms, Alzheimer’s disease

## Abstract

**Background:**

The sortilin-related receptor 1 (*SORL1*) gene, regulating the trafficking and recycling of amyloid precursor protein, has been related to Alzheimer’s disease (AD) and mild cognitive impairment (MCI). The aim of the present study was to investigate the relationship between *SORL1* polymorphisms, plasma concentrations of amyloid-beta (Aβ) isoforms, and AD and MCI susceptibility for a Han Chinese population in Taiwan.

**Methods:**

Eight single-nucleotide polymorphisms (SNPs) in *SORL1* and the apolipoprotein E gene (*APOE*) ε4 alleles were genotyped in 798 patients with AD, 157 patients with MCI, and 401 control subjects. Plasma concentrations of Aβ42, Aβ40, and neuropsychiatric tests for six different cognitive domains were examined.

**Results:**

Among the eight tested SNPs, *SORL1* rs1784933 was most significantly associated with AD and MCI in our population. The G allele of rs1784933 exerted a protective effect and was associated with a reduced risk of AD (odds ratio [OR] = 0.75, *p* = 0.004) and MCI (OR = 0.69, *p* = 0.013). The significance remained after we adjusted for age, sex, and *APOE* ε4 alleles. For the overall participants, the plasma concentrations of Aβ42 were nominally significant for subjects carrying the rs1784933 G allele having a lower level than those without the G allele (*p* = 0.046). There was a similar trend for the G allele carriers to have a lower plasma Aβ40 level than noncarriers, but this was not significant. The nonsynonymous SNP rs2298813 was also related to a lower disease risk when AD and MCI were combined as a group (OR = 0.76, *p* = 0.035). However, there was no association between *SORL1* genotypes and any of the six cognitive tests.

**Conclusions:**

Findings from our study provide support for the effect of *SORL1* gene on the disease risks and pathognomonic surrogates of AD/MCI. The interaction between *SORL1* polymorphisms and Aβ formation requires further study.

## Background

Alzheimer’s disease (AD) is a complex neurodegenerative disease caused by a combination of genetic and environmental influences. The heritability of AD was estimated to be 58–79% in a twins study [1]; yet, long lists of contributory genes have not been fully elucidated. Mutations in the amyloid precursor protein (*APP*), presenilin 1 (*PSEN1*), and presenilin 2 (*PSEN2*) genes are responsible for autosomal dominant inheritance of AD [2]. Genome-wide association studies (GWAS) identified more than 20 susceptible genes for late-onset Alzheimer’s disease (LOAD), including *BIN1, CR1, CLU, CD33, PICALM,* and *ABCA7* [3–5]. Among them, the apolipoprotein E gene (*APOE*) remains the major genetic risk factor of LOAD by posing a 2.6- to 3.2-fold risk in individuals with one copy of the *APOE* ε4 allele and a 14.9-fold risk in those with two copies of the ε4 allele [6, 7].

Accumulation of amyloid-beta (Aβ) peptide, the neurotoxic proteolytic derivative of APP, is central to the pathogenesis of AD. The causative genes of familiar AD (*APP, PSEN1,* and *PSEN2*) and the strongest genetic contributor to LOAD (*APOE*) are all involved in the production, transport, and clearance of Aβ [2, 8]. The protein encoded by sortilin-related receptor 1 gene (*SORL1*) determines the intracellular fate of APP to be recycled or drifted to the endosome-lysosome pathway to generate Aβ [9, 10]. Aberrant expression of *SORL1* has been implicated in AD pathogenesis because the SORL1 protein was found to be downregulated in the brain tissue of patients with sporadic AD [11]. Rogaeva et al. first illustrated that single-nucleotide polymorphisms (SNPs) located within two clusters of the *SORL1* genome (SNPs 8–10 and SNPs 23–25) were related to LOAD susceptibility [12]. This association was later replicated in several ethnic groups, including white, Japanese, Korean, and Chinese populations [13–16]. Previous studies showed that *SORL1* polymorphisms were related to decreased cerebrospinal fluid (CSF) concentrations of Aβ42 and Aβ40, as well as reduced CSF levels of SORL1 protein [17–19]. However, the relationship between *SORL1* polymorphisms and plasma biomarkers of Aβ has never been investigated. Recent studies demonstrated that *SORL1* polymorphisms predict atrophy of AD-specific brain structure (i.e., hippocampal and parahippocampal gyri) in nondemented elderly persons [20], supporting involvement of *SORL1* in the neurodegeneration of cognition-related regions. Investigating the influence of *SORL1* polymorphisms on these clinical and biological endophenotypes could strengthen their pathogenic role in AD.

The aim of the present study was to elucidate whether *SORL1* polymorphisms confer a risk of LOAD and mild cognitive impairment (MCI) in the Han Chinese population in Taiwan, as well as deciphering its effects on different cognitive domains. The influence of *SORL1* polymorphisms on different Aβ isoforms in blood was also examined to give biological evidence for *SORL1*’s effects.

## Methods

### Subjects

A total of 798 patients with LOAD, 157 patients with MCI, and 401 unrelated healthy control subjects were enrolled from Taipei Veterans General Hospital and Taichung Veterans General Hospital, Taiwan. All participants were of Han Chinese descent and resided in Taiwan. The diagnosis of probable AD was made according to the criteria of the National Institute of Neurological and Communicative Disorders and Stroke/Alzheimer’s Disease and Related Disorders Association [21], and the diagnosis of MCI was made according to the revised 2004 consensus criteria [22]. All participants received a comprehensive assessment, including history query, neurological examinations, laboratory tests, and neuroimaging as diagnostic surveys. The control subjects were volunteers without complaints of cognitive dysfunction recruited from outpatient clinics. The study was approved by the institutional review boards of each hospital. All participants provided informed consent according to our institutional guidelines and the recommendations of the Declaration of Helsinki.

### Cognitive testing

For each participant, the global cognitive performance was assessed using the Mini Mental State Examination (MMSE) [23]. Tests specific to each cognitive domain were performed in patients with AD and patients with MCI, including (1) attention (forward and backward digit span from the Wechsler Memory Scale-IV) [24], (2) memory (12-item word recall test) [25], (3) language and executive function (verbal fluency category test) [26], (4) processing speed (Trail Making Test A) [27], and (5) naming task (Boston Naming Test) [28].

### Genotyping

Genomic DNA was extracted from whole blood using the Gentra Puregene kit according to the manufacturer’s protocols (QIAGEN, Hilden, Germany). The ε2, ε3, and ε4 alleles of *APOE* were determined by two SNPs (rs429358 and rs7412) [29]. Eight *SORL1* SNPs were selected on the basis of (1) rs2070045, rs1699102, rs3824968, rs2282649, and rs1010159 (aka SNP19, 22, 23, 24, and 25 in the original report by Rogaeva et al. [12]) being the top signals related to LOAD in white populations [12, 14]; (2) rs3737529 and rs1784933 being the most significant SNPs in Asian populations [15, 16]; and (3) the nonsynonymous SNP rs2298813 having been demonstrated to increase Aβ production in cellular models [30]. All genotyping reactions were carried out using the TaqMan genotyping assay (Applied Biosystems, Foster City, CA, USA). Polymerase chain reactions were performed using 96-well microplates with an ABI 7500 real-time polymerase chain reaction system (Applied Biosystems). Allele discrimination was achieved by detecting fluorescence using SDS software version 1.2.3 (Applied Biosystems).

### Measurement of plasma Aβ concentrations

Plasma samples were available for 592 patients with LOAD, 119 patients with MCI, and 170 control subjects. Within 30 minutes of collection, plasma samples in ethylenediaminetetraacetic acid-containing tubes were centrifuged at 3000 rpm at 4 °C, and the supernatants were collected, divided into aliquots, and frozen at −80 °C. Plasma concentrations of Aβ40 and Aβ42 were measured using the INNO-BIA plasma Aβ forms immunoassay (Fujirebio, Gent, Belgium) as described previously [31]. In brief, the different Aβ isoforms were captured by a mix of beads selectively coated with three different monoclonal antibodies with affinity for Aβ42, Aβ40, and non-Aβ peptides. The immunoreactivity of Aβ42 and Aβ40 were quantified using the xMAP Technology on the Luminex analytical platform (Luminex, Austin, TX, USA).

### Statistical analysis

Hardy-Weinberg equilibrium tests were conducted for each SNP. An additive model of inheritance was presumed to test the associations among *SORL1* SNPs, LOAD, and MCI. A χ^2^ test was used to compare the genotype distributions between LOAD and control subjects, as well as between MCI and control subjects. Multivariate logistic regression without and with adjustment for age, sex, and *APOE* ε4 allele was further used to estimate the odds ratios (ORs) for the risk alleles.

To explore the influence of *SOLR1* SNPs on AD endophenotypes, MMSE scores and serum Aβ concentrations were compared across different *SORL1* genotypes using one-way analysis of variance (ANOVA). For patients with LOAD and patients with MCI, the influence of *SORL1* genotypes on different cognitive domains was also evaluated using ANOVA. All statistical analyses were performed with PASW Statistics software (version 18.0; SPSS, Chicago, IL, USA) with a *p* value <0.05 set as statistically significant. Linkage disequilibrium (LD) blocks were generated by using Haploview version 5.0 software (Broad Institute, Cambridge, MA, USA) using the “solid spine of LD” method, in which a block was formed if the first and last markers were in strong LD with all intermediate markers. The frequency of each haplotype and comparison of the haplotype distributions between AD plus MCI in combination and the control group were performed using Haploview software version 5.0 [32]. To illustrate the LD conformation and haplotype frequency in white populations, the genotype data of the eight *SORL1* SNPs from a CEU population (i.e., Utah residents with Northern and Western European ancestry) were obtained from the 1000 Genomes Project Browser (http://browser.1000genomes.org/index.html).

## Results

### Associations of *SORL1* SNPs and AD/MCI risk

The demographic data of study participants are shown in Table 1. Eight *SORL1* SNPs were genotyped, namely rs2298813 (SNP13), rs2070045 (SNP19), rs1699102 (SNP22), rs3824968 (SNP23), rs3737529, rs2282649 (SNP24), rs1010159 (SNP25), and rs1784933 (SNP26) (Table 2). The genotype distributions of all SNPs complied with Hardy-Weinberg equilibrium.

**Table 1:** Demographic data

	Control subjects (*n* = 401)	MCI (*n* = 157)	AD (*n* = 798)
Age, years	75.4 ± 9.8	74.2 ± 8.3	79.1 ± 8.2**
Male sex	257 (64.1%)	82 (52.2%)*	411 (51.5%)**
Education level, years	11.1 ± 4.9	10.2 ± 4.7	9.7 ± 4.7**
MMSE score	28.0 ± 2.1	26.0 ± 2.8**	18.3 ± 5.9**
12-item word recall test	**–**	4.5 ± 2.9	1.4 ± 2.2^†^
Forward digit span	**–**	10.0 ± 2.5	8.4 ± 3.0^†^
Backward digit span	**–**	5.7 ± 2.4	3.9 ± 2.1^†^
Verbal fluency test	**–**	10.2 ± 3.0	6.5 ± 3.2^†^
Boston Naming Test	**–**	13.6 ± 1.3	11.4 ± 3.0^†^
Trail Making Test A, seconds	**–**	92.3 ± 56.9	181.8 ± 145.2^†^
*APOE* genotypes			
ε2ε2/ε2ε3/ε3ε3	339 (85.0%)	123 (79.4%)*	504 (63.3%)**
ε2ε4/ε3ε4	59 (14.8%)	28 (18.1%)	269 (33.8%)
ε4ε4	1 (0.3%)	4 (2.6%)	23 (2.9%)

*Abbreviations: MCI* Mild cognitive impairment, *AD* Alzheimer’s disease, *MMSE* Mini Mental State Examination, *APOE* apolipoprotein E gene

Data are presented as count (percent) or mean (SD)

** *p* < 0.01 by χ^2^ test or Student’s *t* test when AD or MCI group was compared with control subjects

* *p* < 0.05 by χ^2^ test or Student’s *t* test when AD or MCI group was compared with control subjects

^†^ *p* < 0.01 by Student’s *t* test when comparing patients with AD with patients with MCI

**Table 2:** Genotype distribution of *SORL1* single-nucleotide polymorphisms among patients with Alzheimer’s disease, patients with mild cognitive impairment, and control subjects

		MM/Mm/mm, *n* (%)			Multivariate regression analysis		
SNP	Allele (M/m)	Control subjects	MCI	AD	MCI vs. control subjects	AD vs. control subjects	AD + MCI vs. control subjects
rs2298813 (SNP13)	G/A	291/90/9 (74.6/23.1/2.3)	134/18/2 (87.0/11.7/1.3)	597/176/12 (76.1/22.4/1.5)	OR = 0.49, *p* = 0.003	OR = 0.91, *p* = 0.455	OR = 0.84, *p* = 0.154
					Adj OR = 0.49, *p* = 0.003	Adj OR = 0.82, *p* = 0.156	Adj OR = 0.76, *p* = 0.035
rs2070045 (SNP19)	G/T	144/186/64 (36.5/47.2/16.2)	54/77/25 (34.6/49.4/16.0)	302/392/99 (38.1/49.4/12.5)	OR = 1.04, *p* = 0.794	OR = 0.89, *p* = 0.204	OR = 0.91, *p* = 0.307
					Adj OR = 1.02, *p* = 0.909	Adj OR = 0.87, *p* = 0.149	Adj OR = 0.90, *p* = 0.256
rs1699102 (SNP22)	C/T	330/61/1 (84.2/15.6/0.3)	129/28/0 (82.2/17.8/0.0)	666/123/7 (83.7/15.5/0.9)	OR = 1.13, *p* = 0.620	OR = 1.08, *p* = 0.637	OR = 1.09, *p* = 0.596
					Adj OR = 1.13, *p* = 0.633	Adj OR = 1.06, *p* = 0.711	Adj OR = 1.07, *p* = 0.687
rs3824968 (SNP23)	A/T	155/184/56 (39.2/46.6/14.2)	61/76/19 (39.1/48.7/12.2)	313/390/88 (39.6/49.3/11.1)	OR = 0.96, *p* = 0.772	OR = 0.93, *p* = 0.409	OR = 0.93, *p* = 0.431
					Adj OR = 0.95, *p* = 0.710	Adj OR = 0.90, *p* = 0.300	Adj OR = 0.92, *p* = 0.375
rs3737529	C/T	239/142/20 (59.6/35.4/5.0)	103/50/4 (65.6/31.8/2.5)	508/257/33 (63.7/32.2/4.1)	OR = 0.77, *p* = 0.120	OR = 0.86, *p* = 0.164	OR = 0.85, *p* = 0.107
					Adj OR = 0.78, *p* = 0.140	Adj OR = 0.82, *p* = 0.073	Adj OR = 0.82, *p* = 0.069
rs2282649 (SNP24)	T/C	148/188/56 (37.8/48.0/14.3)	59/79/18 (37.8/50.6/11.5)	305/391/93 (38.7/49.6/11.8)	OR = 0.94, *p* = 0.659	OR = 0.93, *p* = 0.409	OR = 0.93, *p* = 0.408
					Adj OR = 0.92, *p* = 0.585	Adj OR = 0.90, *p* = 0.297	Adj OR = 0.92, *p* = 0.356
rs1010159 (SNP25)	C/T	155/181/55 (39.6/46.3/14.1)	62/77/18 (39.5/49.0/11.5)	315/386/89 (39.9/48.9/11.3)	OR = 0.95, *p* = 0.702	OR = 0.93, *p* = 0.462	OR = 0.94, *p* = 0.463
					Adj OR = 0.93, *p* = 0.598	Adj OR = 0.91, *p* = 0.322	Adj OR = 0.92, *p* = 0.380
rs1784933 (SNP26)	A/G	178/175/47 (44.5/43.8/11.8)	86/61/10 (54.8/38.9/6.4)	408/326/63 (51.2/40.9/7.9)	OR = 0.69, *p* = 0.012	OR = 0.78, *p* = 0.008	OR = 0.77, *p* = 0.003
					Adj OR = 0.69, *p* = 0.013	Adj OR = 0.75, *p* = 0.004	Adj OR = 0.74, *p* = 0.002

*Abbreviations: AD* Alzheimer’s disease, *MCI* Mild cognitive impairment, *SNP* Single-nucleotide polymorphism, *SORL1* Sortilin-related receptor 1 gene, *M* Major allele, *m* Minor allele, *OR* Odds ratio

Model of inheritance was an additive model. Adjusted ORs and adjusted *p* values were obtained from logistic regression with adjustment of age, sex, and *APOE* ε4 allele

*SORL1* rs1784933 was most significantly associated with LOAD and MCI in our population (Table 2). The minor allele G of rs1784933 appeared to exert a protective effect, with significantly lower frequencies of GG genotype in patients with LOAD (7.9%) and in patients with MCI (6.4%) in comparison with control subjects (11.8%) (Table 2). In regression analysis, the G allele was associated with a reduced risk of MCI and LOAD. After adjustment for age, sex, and *APOE* ε4 allele, the G allele remained a significant predictor of MCI (OR = 0.69, *p* = 0.013) and LOAD (OR = 0.75, *p* = 0.004). Combing patients with AD and patients with MCI revealed a stronger association between *SORL1* rs1784933 and AD spectrum disorder (OR = 0.74, *p* = 0.002).

In addition to rs1784933, the nonsynonymous SNP rs2298813 was also significantly associated with MCI (Table 2). The minor allele A of rs2298813 carried a reduced risk of MCI (OR = 0.49, *p* = 0.003) and remained significant after adjustment for age, sex, and *APOE* ε4 allele. The A allele of rs2298813 showed a similar trend, with a protective effect on LOAD, but this result was insignificant. When patients with AD and patients with MCI were combined, the minor allele A of rs2298813 was significantly associated with a reduced risk after adjustment for other covariates (OR = 0.76, *p* = 0.035).

### Haplotype analysis

**Fig. 1:**
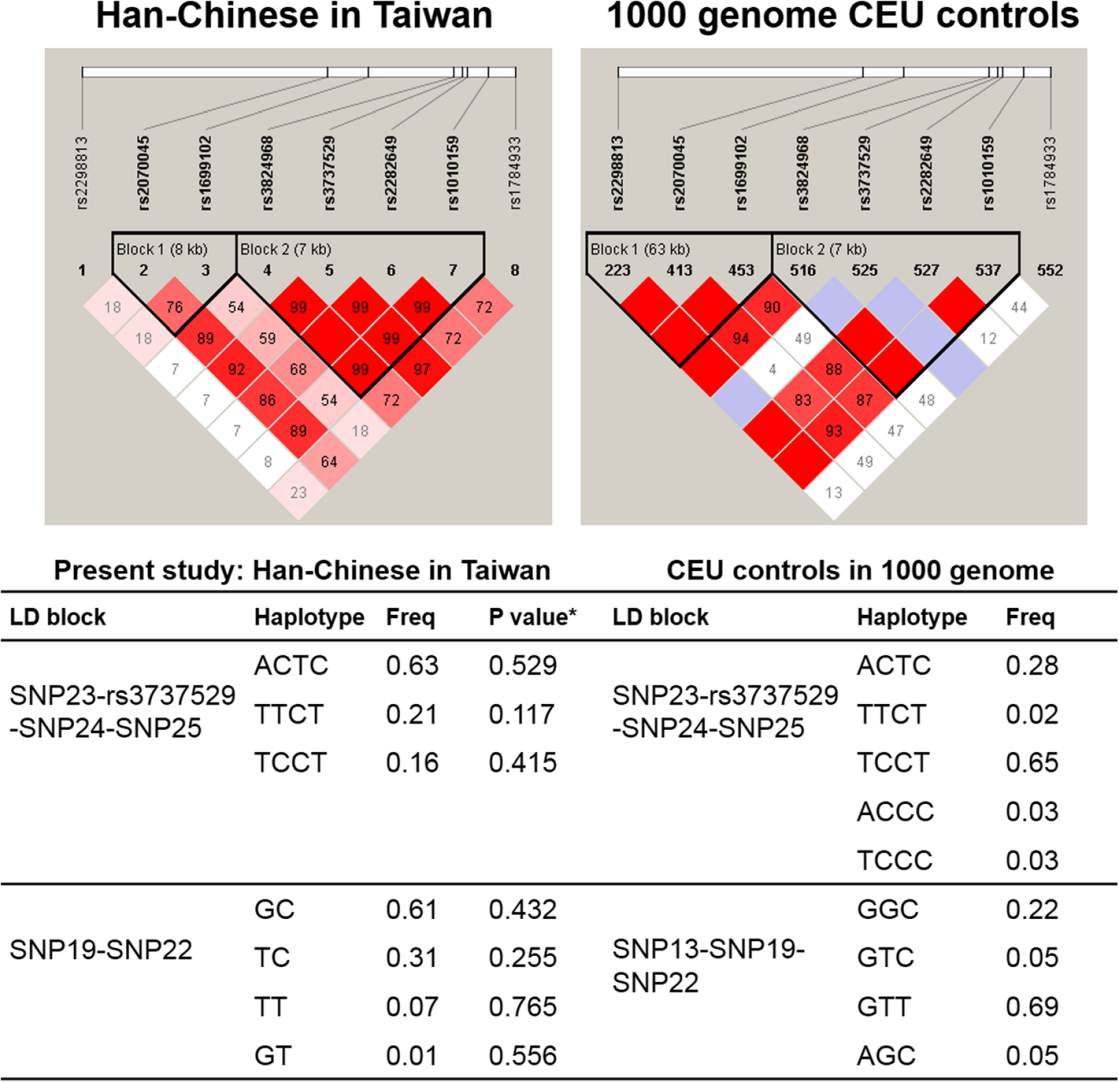
Linkage disequilibrium (LD) map and haplotype analysis. * *p* value for comparing haplotype distribution between patients with AD + patients with MCI and control subjects using Haploview software version 5.0. The frequency (Freq) of each haplotype and the conformation of LD blocks differed substantially between Han Chinese in Taiwan and a white population (CEU; Utah residents with Northern and Western European ancestry) from the 1000 Genomes Project. *SNP* Single-nucleotide polymorphism

LD mapping of the eight genotyped *SORL1* SNPs showed that there were two LD blocks in the Han Chinese population in Taiwan (Fig. 1, *left panel*). One LD block was composed of rs2070045 and rs1699102 (SNPs 19–22), and the other one included four SNPs (rs3824968, rs3737529, rs2282649, and rs1010159; SNPs 23–rs3737529–25). Haplotype analysis was performed in the two LD blocks separately, but it failed to yield any significant result (Fig. 1). When comparing the LD maps between the Han Chinese population in the present study and the CEU population from the 1000 Genomes Project, the LD conformation and the haplotype frequency substantially differed between the two ethnic groups. For example, “ACTC” was the most common haplotype of the second LD block (SNPs 23–rs3737529–SNP25) in the Han Chinese population, but “TCCT” was the most common one in the CEU population (Fig. 1).

### Associations of *SORL1* SNPs and plasma Aβ concentrations

We further explored the relationship between rs1784933 genotypes and plasma Aβ concentrations (Table 3). The average plasma concentrations of Aβ40 and Aβ42, as well as the ratios of plasma Aβ42/Aβ40 concentration, were similar among control subjects, patients with MCI, and patients with LOAD. Overall, the plasma concentrations of Aβ40 and Aβ42 were significantly lower in subjects carrying the rs1784933 G allele than among those without that G allele. After adjusting for age, sex, and *APOE* ε4 allele, the association of the G allele of *SORL1* rs1784933 with a lower plasma concentration of Aβ42 remained nominally significant (*p* = 0.046); however, the results were only borderline significant for a lower plasma concentration of Aβ40 (*p* = 0.071). In addition, the ratio of plasma Aβ42/Aβ40 concentration did not differ between rs1784933 G allele carriers and noncarriers. Similar analysis done for patients with LOAD alone revealed lower plasma Aβ42 concentrations in G allele carriers than in noncarriers. However, the plasma concentration of Aβ40 and the ratio of Aβ42/Aβ40 concentration in patients with LOAD did not show any difference between G allele carriers and noncarriers.

**Table 3:** Associations between *SORL1* rs1784933 and plasma amyloid-beta concentration

	Multivariate regression model	Aβ42	Aβ40	Aβ42/Aβ40 ratio
AD (*n* = 592)		23.8 ± 15.1	173.1 ± 79.3	0.15 ± 0.25
MCI (*n* = 119)		23.6 ± 12.5	178.7 ± 54.6	0.14 ± 0.07
Control subjects (*n* = 170)		23.7 ± 12.6	171.6 ± 64.3	0.15 ± 0.08
AD vs. control subjects		*p* = 0.899	*p* = 0.807	*p* = 0.904
MCI vs. control subjects		*p* = 0.969	*p* = 0.318	*p* = 0.189
*SORL1* rs1784933 in overall participants (*n* = 873)				
G allele carriers (AG + GG genotypes)		22.68 ± 13.82	168.51 ± 65.93	0.14 ± 0.08
G allele noncarriers (AA genotype)		24.82 ± 14.67	178.33 ± 79.81	0.16 ± 0.27
G allele carriers vs. noncarriers				
	Raw *p* value	*p* = 0.026	*p* = 0.048	*p* = 0.214
	Adjusted for age and sex	Adj *p* = 0.029	Adj *p* = 0.054	Adj *p* = 0.217
	Adjusted for age, sex, and *APOE* ε4 allele	Adj *p* = 0.046	Adj *p* = 0.071	Adj *p* = 0.248
*SORL1* rs1784933 in patients with AD (*n* = 584)				
G allele carriers (AG + GG genotypes)		22.62 ± 14.48	168.86 ± 70.79	0.14 ± 0.08
G allele noncarriers (AA genotype)		25.00 ± 15.60	177.14 ± 86.24	0.16 ± 0.33
G allele carriers vs. noncarriers				
	Raw *p* value	*p* = 0.056	*p* = 0.205	*p* = 0.203
	Adjusted for age and sex	Adj *p* = 0.056	Adj *p* = 0.204	Adj *p* = 0.203
	Adjusted for age, sex, and *APOE* ε4 allele	Adj *p* = 0.058	Adj *p* = 0.211	Adj *p* = 0.206

*Abbreviations: AD* Alzheimer’s disease, *APOE* Apolipoprotein E gene, *MCI* Mild cognitive impairment, *SORL1* Sortilin-related receptor 1 gene, *Aβ* Amyloid-beta, *Adj p p* value in the multivariate regression with adjustment of covariates

### Associations of *SORL1* SNPs and cognitive tests

To further test the influence of rs1784933 genotypes on cognitive function, the average MMSE scores among the three genotypes were compared in patients with LOAD, patients with MCI, and control subjects separately. There was no significant difference in the MMSE scores among the AA, AG, and GG genotypes of rs1784933 (Table 4). For patients with MCI and patients with LOAD, there was no association between rs1784933 genotypes and any of the six cognitive test results (Table 4). Neither the MMSE scores nor any of the six cognitive domain tests showed differences across rs2298813 genotypes (data not shown).

**Table 4:** Associations between *SORL1* rs1784933 and cognitive tests

Subjects	Cognitive tests	*SORL1* rs1784933			*p* Value (ANOVA)
		AA	AG	GG	
Control subjects	MMSE score	28.0 ± 2.1	28.1 ± 1.9	27.6 ± 2.1	0.490
MCI	MMSE score	25.9 ± 2.9	26.0 ± 2.8	26.7 ± 2.4	0.468
	12-item word recall test	4.5 ± 3.0	4.5 ± 2.7	5.0 ± 2.6	0.877
	Forward digit span	10.0 ± 2.8	10.2 ± 2.1	9.1 ± 2.9	0.507
	Backward digit span	5.5 ± 2.5	6.1 ± 2.1	6.3 ± 3.2	0.271
	Verbal fluency test	10.1 ± 2.8	10.5 ± 3.4	9.5 ± 2.3	0.576
	Boston Naming Test	13.5 ± 1.2	13.8 ± 1.3	13.4 ± 1.4	0.290
	Trail Making Test A, seconds	99.7 ± 67.2	84.1 ± 38.9	74.4 ± 39.0	0.177
AD	MMSE score	18.1 ± 6.0	18.5 ± 5.7	18.5 ± 6.0	0.648
	12-item word recall test	1.3 ± 2.1	1.5 ± 2.2	1.5 ± 2.5	0.494
	Forward digit span	8.3 ± 3.1	8.6 ± 3.1	8.1 ± 2.2	0.224
	Backward digit span	3.8 ± 2.1	3.9 ± 2.2	3.5 ± 1.8	0.461
	Verbal fluency test	6.4 ± 3.3	6.5 ± 3.1	6.9 ± 2.8	0.537
	Boston Naming Test	11.2 ± 3.1	11.5 ± 2.9	11.9 ± 2.3	0.229
	Trail Making Test A, seconds	186.8 ± 160.5	175.6 ± 130.4	182.9 ± 115.2	0.630

*Abbreviations: AD* Alzheimer’s disease, *MCI* Mild cognitive impairment, *SORL1* Sortilin-related receptor 1 gene, *MMSE* Mini Mental State Examination, *ANOVA* One-way analysis of variance

## Discussion

The present study confirmed *SORL1* as a susceptible gene for LOAD and MCI in the Han Chinese population in Taiwan. The SNP rs1784933 located in the 3′ region of the *SORL1* genome and the nonsynonymous SNP rs2298813 were most significantly associated with AD and MCI. A lower plasma level of Aβ42 was found in individuals carrying the minor allele G of rs1784933 in comparison with those without the G allele. A similar trend of reduced plasma levels of Aβ40 was also observed in the G allele carriers, but this finding was not significant. Neither MMSE scores nor any test of the six cognitive domains differed among *SORL1* genotypes.

In the Taiwanese population, SNP rs1784933 (SNP26) is most significantly associated with AD/MCI susceptibility, and its minor allele G exerts a protective effect against disease. Consistent with our findings, in a study of persons of Han Chinese descent in mainland China, researchers found that the G allele of rs1784933, but not the other two tested *SORL1* SNPs was related to a reduced risk of AD [16]. Although the associations between *SORL1* polymorphisms and AD have been replicated in several studies [14, 15], the regions tagged by most significant SNPs vary across different ethnic groups. For whites, Caribbean Hispanics, and Israeli Arabs, SNPs located in the 5′ end of the *SORL1* genome (i.e., SNPs 8–10) are most strongly associated with AD [12, 14]. However, SNPs near the 3′ region of the *SORL1* genome (i.e., SNP19 and SNPs 22–25) are more significantly related to AD in the Chinese, Japanese, and African American populations [13, 33, 34]. The consistent findings between our study and other Asian groups imply a pathogenic role of the 3′ region of *SORL1* in AD, especially for Asian populations. In addition, the different haplotype frequency and LD conformation between Han Chinese and CEU populations (Fig. 1) further explain why the most significant SNPs vary across populations.

It is worthwhile to note that the nonsynonymous SNP rs2298813 (A528T), causing an amino acid substitution from alanine to threonine at the 528th residue of SORL1 protein, was significantly associated with MCI in our population. A similar but insignificant effect of rs2298813 on LOAD was also observed. Interestingly, rs2298813 was rarely found significant in previous GWAS of LOAD, but this coding variant segregates with disease status in familial AD [30]. The results of an in vitro study suggest that this coding variant has a direct and deleterious impact on AD pathogenesis because HEK293 cells expressing A528T mutant SORL1 could not physiologically interact with APP, which subsequently increased the secretion of Aβ42, soluble APPα, and APPβ [30].

The SORL1 protein regulates APP trafficking and processing, which subsequently influences the formation of Aβ [9]. Researchers in several studies explored the relationship between *SORL1* polymorphisms and CSF levels of Aβ42 and Aβ40, but their work led conflicting results [17–19, 35, 36]. Concordant with our findings that subjects carrying the minor allele of rs1784933 have reduced plasma levels of Aβ42, investigators in several studies found that SNPs located at the 3′ region of *SORL1* were associated with lower concentrations of Aβ42 in CSF [17–19]. A trend for reduced CSF concentrations of Aβ40 was also observed in these studies, but without statistical significance. Because SORL1 regulates the APP processing pathway upstream from the catalyzation of β- and γ-secretases, insufficient SORL1 activity would not change the ratio of Aβ42/Aβ40 concentrations.

To our knowledge, the present study is the first investigation of the influence of *SORL1* polymorphisms on plasma concentrations of Aβ42 and Aβ40. Although plasma Aβ concentrations might be confounded by age, disease duration, and other factors [37, 38], they are more easily accessible than CSF Aβ levels as a surrogate marker of AD pathology. Notably, the association between plasma Aβ levels and *SORL1* rs1784933 derived mainly from patients with AD rather than from patients with MCI (Table 3). There might be two reasons for such a discrepancy. First, only 30–60% of patients with MCI have a neurodegenerative and progressive course, with the remainder having nondegenerative (or reversible) causes [39, 40]. The MCI group consists of heterogeneous entities, including AD and other pathogenesis, which might account for the insignificant correlation between *SORL1* polymorphisms and plasma Aβ concentrations. Second, the smaller sample size of the patients with MCI with available plasma Aβ levels may have limited our power to detect a significant correlation. We did not measure the plasma concentrations of SORL1 protein, because it is undetectable in the circulation, according to a previous study [41].

The relationship between *SORL1* variants and cognitive function has been investigated. Reynolds et al. found that markers at the 5′ region of *SORL1* tended to be associated with verbal function decline and that SNPs near the 3′ end were more related with episodic memory impairment [42]. However, in a large cohort with a sample size up to 9624 participants, researchers did not find any correlation between *SORL1* variants and different domains of cognitive function [43]. The present study also does not demonstrate any association between *SORL1* SNPs and the six cognitive domains.

## Conclusions

*SORL1* is genetically related to MCI and LOAD in the Han Chinese population in Taiwan. A reduced plasma concentration of Aβ42 was found in individuals carrying the minor allele of the most significant SNP, rs1784933, implying a biological role of *SORL1* genetic markers on the Aβ cascade.

## Abbreviations

AD: Alzheimer’s disease
ANOVA: Analysis of variance
*APOE*: Apolipoprotein E gene
*APP*: Amyloid precursor protein gene
Aβ: Amyloid-beta
CEU: Utah residents with Northern and Western European ancestry
CSF: Cerebrospinal fluid
GWAS: Genome-wide association studies
LD: Linkage disequilibrium
LOAD: Late-onset Alzheimer’s disease
MCI: Mild cognitive impairment
MMSE: Mini Mental State Examination
*PSEN1*: Presenilin 1 gene
*PSEN2*: Presenilin 2 gene
SNP: Single-nucleotide polymorphism
*SORL1*: Sortilin-related receptor 1 gene
